# Prevalence and associated factors in burnout and psychological morbidity among substance misuse professionals

**DOI:** 10.1186/1472-6963-8-39

**Published:** 2008-02-08

**Authors:** Adenekan Oyefeso, Carmel Clancy, Roger Farmer

**Affiliations:** 1 Division of Mental Health, Medical School, St George's, University of London, London SW17 0RE, UK; 2 School of Health and Social Sciences, Middlesex University, F Block, Holborn Union Building, Archway Campus, Highgate Hill, London N19 3UA, UK; 3 South West London and St George's Mental Health NHS Trust, Richmond Royal Hospital, Kew Foot Road, Surrey TW9 2TE, UK

## Abstract

**Background:**

Studies of psychological stress among substance misuse professionals rarely describe the nature of burnout and psychological morbidity. The main aim of this study was to determine the extent, pattern and predictors of psychological morbidity and burnout among substance misuse professionals.

**Methods:**

This study was a cross-sectional mail survey of 194 clinical staff of substance misuse services in the former South Thames region of England, using the General Health Questionnaire (GHQ-12) the Maslach Burnout Inventory (MBI) as measures of psychological morbidity and burnout, respectively.

**Results:**

Rates of psychological morbidity (82%: 95% CI = 76–87) and burnout (high emotional exhaustion – 33% [27–40]; high depersonalisation – 17% [12–23]; and diminished personal accomplishment – 36% [29–43]) were relatively high in the study sample. High levels of alienation and tension (job stressors) predicted emotional exhaustion and depersonalisation (burnout) but not psychological morbidity. Diminished personal accomplishment was associated with higher levels of psychological morbidity

**Conclusion:**

In the sample of substance misuse professionals studied, rates of psychological morbidity and burnout were high, suggesting a higher level of vulnerability than in other health professionals. Furthermore, pathways to psychological morbidity and burnout are partially related. Therefore, targeted response is required to manage stress, burnout and psychological morbidity among substance misuse professionals. Such a response should be integral to workforce development.

## Background

Since the introduction of the United Kingdom Government's Drug Strategy in 1998, substance misuse services have expanded with increases in funding available from central government as part of implementation of the drug strategy [1]. The targets set in the strategy may have put extra demands on substance misuse services with a likely increase in job-related stress, burnout and associated psychological morbidity.

Studies of stress and burnout in various occupational groups and settings have been widely reported [2-4]. However, few studies have examined burnout in substance misuse workers. An earlier study of burnout among substance misuse workers in the UK [5] revealed high emotional exhaustion and high depersonalisation in this population. In addition, very few studies have examined work-related predictors of burnout in substance misuse workers, and these have been limited to the addiction workforce in the United States [6,7]. Similarly, many studies have been conducted on the prevalence and pattern of psychological morbidity in different occupational groups and settings [8-10]. Yet, very few have focused on psychological morbidity and its predictors in substance misuse professionals.

There are pointers in the literature to the presence of high occupational stress burnout and high psychological morbidity among substance misuse professionals. Human services, such as substance misuse practice, that entail relatively low practitioner autonomy tend to be strongly associated with high psychological morbidity [11]). Secondly, substance misuse practice has been associated with high demands and low control over caseload and tasks [5]. These circumstances are similar to the concept of job strain that has been articulated by Karasek et al [12]. Furthermore, Calnan et al [13] have demonstrated a strong relationship between job strain and psychological morbidity.

Determining the extent, pattern and predictors of burnout and psychological morbidity among substance misuse professionals can lead to major benefits such as:

• Improving job satisfaction and retention in the workforce, given the significant negative relationship between stress and job satisfaction

• Providing information that should assist employee support and the development of programmes to promote employee well-being

• Helping employers address employee mental health needs with a view to improving overall psychological health and job performance.

The aim of this study was to determine the prevalence, pattern and predictors of burnout and psychological morbidity using data collected during the earlier stages of implementation of the Government's ten-year drug strategy.

The study objectives were to determine the prevalence of burnout and psychological morbidity among substance misuse service workers; the influence of demographic variables, job characteristics and job stressors on burnout and psychological morbidity; and examine the relationship between burnout and psychological morbidity.

## Methods

This study was designed to test the following hypotheses:

1. Age and gender would predict burnout and psychological morbidity.

2. Job characteristics would predict burnout and psychological morbidity.

3. Job stressors would predict burnout and psychological morbidity.

4. There would be a significant positive relationship between burnout and psychological morbidity.

Maslach and Jackson's [14] definition of burnout was adopted in this study. However, the three dimensions of burnout, emotional exhaustion, depersonalisation and diminished were examined separately. Psychological morbidity was defined as scores on the General Health Questionnaire – 12 (GHQ-12) [15].

The data reported in this article were collected as part of a cross-sectional postal survey of clinical staff of substance misuse services in the former South Thames region of England in 2000. Staff from private clinics were excluded from this analysis. The survey questionnaire covered many areas including demographic details, job characteristics, measures of burnout, job stressors, visual analogue scales of job stress and job satisfaction and psychological morbidity. The relationships between job stress, burnout and job satisfaction have been reported previously in the development and validation of an occupational stress scale among substance misuse professionals [16].

### Subjects

The sample consisted of clinical staff working in substance misuse services (statutory and non-statutory) in the former South Thames (West) region of England. The sampling frame was based on the number of services listed in the directory of substance misuse services published by health authorities. Secondly, the manager of each service was requested to provide the number of current staff with existing caseload. This mapping exercise yielded 280 staff that were surveyed from 46 services. Staff from these services provided a sample size of 194, yielding a response rate of 69% (the number of respondents returning a questionnaire as a percentage of all identified clinical staff in participating agencies), after a second wave that involved a telephone reminder. The first wave of the postal survey yielded a response rate of 52% after one month. We were unable to determine the nature and magnitude of non-response bias because at the time of the study, there was no information on the characteristics of substance misuse clinical staff in the region.

The mean age of respondents was 38 years (SD = 9.9). Participants were 57% female and the following occupational groups were represented: Nurses (36%, n = 70); drug/alcohol counsellors (29%, n = 56); social workers (8%, n = 15); doctors (6%, n = 12); clinical psychologists (3%, n = 6); and others (e.g., occupational therapist, probation officers, outreach workers, drug support workers, etc: 18%, n = 35).

### Dependent variables

The four dependent variables were emotional exhaustion (EE); depersonalisation (DP) and diminished personal accomplishment (PA) and psychological morbidity (PM). The three dimensions of burnout were measured with the Maslach Burnout Inventory (MBI) [17]. Using the norm reported in the manual [17], respondents with the following scores were classified as 'high' scorers and, therefore fulfilled the criteria for burnout: EE ≥ 27; DP ≥ 13; and PA ≤ 31. Psychological morbidity was measured with the general health questionnaire-12 (GHQ-12), scored using the 0-0-1-1 scoring format with scores ranging from 0–12. Caseness for psychological morbidity was determined using a cut-off of 4 [18]. Both measures are widely used instrument for measuring burnout and psychological morbidity, respectively.

### Independent variables

The independent variables were demographic characteristics; job characteristics and job stressors. The demographic variables included in the analysis were age and gender. Job characteristics were intensity of client contact (ICC: number of hours of weekly contact) and tenure (number of years of experience in substance misuse). Participants were asked to indicate the extent to which a list of 112 job stressors, obtained from the literature and from discussions with a sample of clinical staff, gave them pressure using a Likert-type scale (no pressure, slight pressure; moderate pressure, considerable pressure, extreme pressure).

### Job stressors

Participants' response to the questions on job stressors was subjected to internal consistent analysis (Cronbach α). Items that resulted in a decrease in α were excluded from further analysis. This procedure yielded 68 internally consistent items.

Principal component analysis, with varimax rotation, was used to reduce the number of internally consistent job stressors experienced by respondents to manageable types or factors. The Scree test was used to determine the number of factors (or types). A stressor belonged to a factor if it returned a factor loading ≥ 0.40. Furthermore, a job stressor was excluded from the rotated factors if it had a factor loading ≥ 0.40 loaded on two or more factors [19].

The principal component analysis yielded three orthogonal factors. Factor 1 termed 'Alienation' consisted of 15 stressors. Examples of stressors in this factor were "Lack of support from senior staff"; "Feelings of isolation"; and "Role ambiguity." Factor 2, termed 'Case complexity', consisted of 13 stressors with the following examples: "Manipulative clients"; "Demanding clients"; and "Dealing with clients with overdose." Factor 3, termed 'Tension" consisted of 15 stressors such as "Conflicting demands of my time at work by others"; Having too little time to do what is expected of me"; and "Work overload" (Additional file 1).

The three job stressor variables were categorised into two levels. Participants whose scores were greater than or equal to the mean on each factor were classified as experiencing high levels of Alienation, Case complexity and Tension, respectively. Participants that score below the mean were classified as experiencing low levels of each category of job stressor.

### Statistical analysis

Cronbach's α was used to assess internal consistency of validated measures – MBI and GHQ-12 (Likert-type scale).

Prior to logistic regression analysis, univariate odds ratio was used to determine the relationship between categorical independent and dependent variables. The relationship between interval independent variables (ICC and tenure) and categorical dependent variables was determined using point-biserial correlation. Logistic regression analysis was used to examine the association between independent and the dependent variables. Using the Hosmer and Lemeshow [20] criterion, an independent variable was included in the logistic regression model if the univariate odds ratio or point-biserial correlation had a p value of 0.1 or less.

Dummy variables of occupational groups, with nurses as the referent variable, were developed and introduced into logistic regression as control variables. A p-value of < 0.05 was considered to indicate statistical significance. SPSS version 15 was used for all statistical analyses.

### Ethics approval

The Wandsworth Local Research Ethics Committee approved this study.

## Results

### Internal consistency of measures

The four dependent measures in the study had acceptable internal consistency in the study sample: GHQ-12, Cronbach's α = 0.75; MBI EE subscale, α = 0.90; DP subscale, α = 0.76; and PA subscale, α = 0.75.

### Prevalence of psychological morbidity

The prevalence rate of psychological morbidity in the sample was 82.3% (95% CI = 76.1–87.4). Male and female prevalence rates were 82.4% (95% CI = 72.6–89.8) and 82.2% (73.7–89.0), respectively.

### Prevalence of burnout

The rate of burnout was as follows: high emotional exhaustion (EE), 33.2% (95% CI = 26.5–40.4); high depersonalisation (DP), 17.0% (95% CI = 11.9–23.2); and diminished personal accomplishment (PA), 35.8% (95% CI = 29.0–43.2). Male and female burnout rates were as follows: Male high EE rate, 31.7% (95% CI = 21.9–42.9); female high EE rate 34.3% (95% CI = 25.3–44.2); male high DP rate, 20.7% (95% CI = 12.6–31.1); female high DP rate, 14.2% (95% CI = 8.1–22.3); male low PA rate, 29.3% (95% CI = 19.7–40.4); and female low PA rate, 41.0% (95% CI = 31.5–51.0).

### Summary of dependent measures

There was no significant difference between occupational groups on all dependent measures (Table 1).

**Table 1:** Summary statistics of dependent measures by occupational group

Occupation	GHQ-12*	EE	DP	PA
Nurses				
Mean	5.6	21.9	8.2	33.0
SD	2.6	9.8	5.9	7.7
N	68	68	69	69

Social workers				
Mean	71	26.1	9.2	33.7
SD	2.5	13.5	5.8	5.1
N	15	15	15	15

Doctors				
Mean	6.4	19.3	6.6	30.7
SD	2.2	8.8	4.8	9.2
N	11	11	10	10

Clinical psychologists				
Mean	5.7	29.4	6.2	35.8
SD	3.1	8.6	7.5	9.0
N	6	5	5	6

Drug and alcohol counsellors				
Mean	5.7	21.4	6.1	35.4
SD	2.8	10.6	5.4	6.3
N	54	54	54	53

Other				
Mean	6.1	21.7	7.7	33.1
SD	2.9	11.9	5.8	7.2
N	33	34	34	34

F statistic	0.9 (p = 0.48)	1.1 (p = 0.37)	1.2 (p = 0.31)	1.2 (p = 0.30)

* Scoring: 0-0-1-1

### Selection of potential predictors of psychological morbidity

Univariate odds ratio, using the Hosmer & Lemeshow criterion (p < = 0.1) revealed acceptable correlation coefficients between psychological morbidity (PM) and Alienation (p = 0.07); Tension (p = 0.07); EE (0.02); DP (0.1); and PA (0.009. Point-biserial correlation analysis revealed significant positive relationship between PM and age (p = 0.1); intensity of client contact (ICC: p = 0.07); and tenure (0.05). These variables were included in the logistic regression model.

### Selection of potential predictors of EE, DP and PA

Correlation between EE and the following variables met the criterion for selection: Tension (p = 0.0001); Alienation (p = 0.0001); Case complexity (p = 0.04); and age (p = 0.01). Correlation between DP and Tension (p = 0.0001); Alienation (p = 0.004); Case complexity (p = 0.001); age (p = 0.02); and tenure (p = 0.1) met the criterion for selection. Finally, correlation between PA, age (p = 0.1); and gender (p = 0.07) met the criterion for selection.

Five logistic regression models emerged from the findings of the univariate analysis. These models were adjusted for occupational groups and used to test the study hypotheses:

*1. Log [P(PM = 1)/P(PM = 0)] = b*_0_*+ b*_1_*Tension + b*_2_*Age + b*_3_*ICC + b*_4_*Tenure*.

*2. Log [P(PM = 1)/P(PM = 0)] = b*_0 _*+ b*_1_*EE + b*_2_*DP +b*_3_*PA*.

*3. Log [P(EE = 1)/P(EE = 0)] = b*_0_*+ b*_1_*Tension + b*_2_*Alienation + b*_3_*Case complexity +b*_4_*Age + b*_5_*Tenure*.

*4. Log [P(DP = 1)/P(DP = 0)] = b*_0_*+ b*_1_*Tension + b*_2_*Alienation + b*_3_*Case complexity + b*_4_*Age + b*_5_*Tenure*.

*5. Log [P(PA = 1)/P(PA = 0)] = b*_0_*+ b*_1_*Age + b*_2_*Gender*.

The variables were coded as follows: Gender (0 = Male, 1 = Female); age (dummy variables were developed for under-25s; 25–34; 35–44; referent = 45 and over); ICC (categorised as low – below the mean = 0; and high – mean and above = 1); Tenure (categorised as short – below the mean = 0; and long – mean and above = 1); Alienation (0 = low, 1 = high); Case Complexity (0, low, 1 = high); Tension (0 = low, 1 = high); PM (0 = low, 1 = high); EE (0 = low, 1 = high); DP (0 = low, 1 = high);and PA (0 = high, 1 = low).

### Predictors of psychological morbidity and burnout

The first hypothesis, which predicted that age and gender would predict psychological morbidity and burnout, was partially confirmed. Gender did not predict psychological morbidity and the three dimensions of burnout. However, age was a significant predictor of emotional exhaustion. Compared to those aged 45 years and over, participants aged below 25 years were seven times as likely to experience high emotional exhaustion. However, there was no association between age and psychological morbidity and other dimensions of burnout (DP and PA) (Table 2).

**Table 2:** Logistic regression models for psychological morbidity and burnout

**Dependent variable**	**Multivariate predictors**	**Wald χ^2^**	**P value**	**Adjusted OR**	**95% CI**
**Model 1**					
Psychological morbidity (GHQ-12)	High level of Alienation	1.50	0.22	2.00	0.66–6.11
	High level of Tension	0.13	0.72	1.19	0.47–6.11
	Age: Under 25s	0.51	0.48	2.32	0.23–23.23-
	Age: 25–34 years	1.40	0.24	0.53	0.18–1.52
	Age: 35–44 years	0.04	0.85	0.90	0.30–2.69
	Age: 45 year and over	-	-	1.00	-
	Long tenure	0.56	0.46	1.39	0.59–3.30
	High ICC	0.38	0.54	1.30	0.57–2.95
	Social worker	2.04	0.15	4.95	0.55–44.28
	Doctor	0.60	0.44	2.40	0.26–21.96
	Clinical psychologist	0.02	0.89	1.19	0.11–12.64
	Drug and alcohol counsellor	0.14	0.71	1.22	0.44–3.35
	Other	0.44	0.51	0.70	0.24–2.03
	Nurse	-	-	1.00	-

**Model 2**					
Psychological morbidity (GHQ-12)	High emotional exhaustion	3.47	0.06	2.83	0.95–8.43
	High depersonalisation	0.11	0.74	1.28	0.31–5.35
	**Diminished personal accomplishment**	**6.61**	**0.01**	**3.65**	**1.36–9.79**
	Social worker	1.11	0.29	3.22	0.37–28.19
	Doctor	0.66	0.42	2.50	0.28–22.72
	Clinical psychologist	0.004	0.95	0.93	0.09–10.19
	Drug and alcohol counsellor	0.48	0.49	1.41	0.53–3.76
	Other	1.15	0.28	0.57	0.20–1.59
	Nurse	-	-	1.00	-

**Model 3**					
Burnout: Emotional exhaustion	**High levels of Alienation**	**7.54**	**0.006**	**3.49**	**1.43–8.51**
	**High levels of Tension**	**4.46**	**0.04**	**2.65**	**1.10–6.52**
	High levels of Case complexity	0.04	0.85	1.10	0.41–2.94
	Long tenure	0.002	0.96	1.02	0.43–2.40
	**Age: Under 25s**	**4.43**	**0.04**	**7.15**	**1.15–44.65**
	Age: 25–34 years	1.55	0.21	2.02	0.67–6.11
	Age: 35–44 years	0.62	0.43	1.54	0.53–4.45
	Age: 45 & over	-	-	1.00	-
	Social worker	5.53	0.02	4.82	1.30–17.87
	Doctor	0.03	0.87	0.86	0.14–5.46
	Clinical psychologist	0.20	0.67	1.58	0.21–11.68
	Drug and alcohol counsellor	0.07	0.79	1.15	0.42–3.10
	Other	2.07	0.15	2.18	0.75–6.312
	Nurse			1.00	

**Model 4**					
Burnout: Depersonalisation	High levels of Alienation	1.30	0.25	1.87	0.64–5.48
	**High levels of Tension**	**5.68**	**0.02**	**4.57**	**1.31–15.91**
	High levels of Case complexity	1.85	0.17	2.07	0.73–5.86
	Long tenure	2.31	0.13	0.43	0.15–1.27
	Age: Under 25s	2.25	0.13	5.45	0.60–48.90
	Age: 25–34 years	0.92	0.34	2.14	0.45–10.14
	Age: 35–44 years	1.78	0.18	2.78	0.62–12.45
	Social worker	0.05	0.83	1.22	0.20–7.34
	Doctor	0.56	0.45	0.38	0.03–4.73
	Clinical psychologist	0.72	0.40	0.33	.003–4.25
	Drug and alcohol counsellor	2.11	0.15	0.32	0.07–1.49
	Other	0.03	0.86	1.12	0.33–3.84
	Nurse	-	-	1.00	-

**Model 5**					
Burnout: Diminished Personal accomplishment	Gender	2.42	0.12	1.67	0.88–3.18
	Age: Under 25s	0.70	0.40	1.86	0.43–7.94
	Age: 25–34 years	0.01	0.93	1.04	0.47–2.27
	Age: 35–44 years	0.12	0.73	0.86	0.37–2.02
	Age: 45 and over	-	-	1.00	-
	Social worker	2.43	0.12	0.34	0.09–1.32
	Doctor	0.002	0.97	1.03	0.26–4.09
	Clinical psychologist	0.18	0.67	0.68	0.11–4.05
	Drug and alcohol counsellor	3.00	0.08	0.49	0.22–1.10
	Other	0.02	0.90	0.95	0.40–2.22
	Nurse	-	-	1.00	-

As stated in the second hypothesis, there was no evidence that job characteristics (tenure and ICC) predicted psychological morbidity and burnout. However, the third hypothesis, which stated that job stressors would predict psychological morbidity and burnout, was partially supported. High scorers on alienation and tension were thrice as likely to experience emotional exhaustion as low scorers on both independent variables. Furthermore, high scorers on alienation were five times as likely to experience depersonalisation as low scorers (Table 2)

The fourth hypothesis that predicted a significant positive relationship between burnout and psychological morbidity was partially confirmed. Diminished personal accomplishment was the only burnout dimension that significantly predicted psychological morbidity. Respondents with diminished personal accomplishment were about four times as likely to experience psychological morbidity (Table 2)

## Discussion

The findings of this study reveal that the prevalence of psychological morbidity among substance misuse workers is high (82%). The prevalence of burnout was not as pronounced, with 33% of participants reporting high EE; 17% reporting high DP; and 36% reporting diminished PA. The average EE, DP and PA scores in the study sample were 22.1, 7.4 and 33.7, respectively. The EE score in our sample was higher than that in most human services occupational groups, e.g., teaching, 21.3; postsecondary education, 18.6; social services, 21.4; and mental health, 16.9; but similar to that in medicine, 22.2 [17]. Our findings, therefore, strongly indicate that substance misuse professionals are more vulnerable to burnout than most human services professionals. Furthermore, compared to nurses, social workers were at higher risk of emotional exhaustion. This is an observation that has not been previously reported among substance misuse professionals.

One of the novel findings in this study is the identification of three types of stressors among substance misuse professionals – Alienation, Tension and Case Complexity. The constructs of alienation and tension are consistent with the Job Demand-Control (JDC) model developed by Karasek [21], while Case Complexity encompasses the Client Demand subscale of the Addiction Employee Stress Scale [16].

Identification of these three categories of stressors is useful for two reasons. It helps to organise the wide range of job stressors linked to substance misuse practice into manageable segments. Secondly, it facilitates better understanding of the link between job stressors and burnout by revealing the types of job stressors that are directly associated with burnout. From this study, alienation and tension emerged as strong predictors of emotional exhaustion and depersonalisation.

Although job stressors (alienation and tension) predicted two dimensions of burnout, none of these factors was directly linked to psychological morbidity. This finding contrasts with that of Calnan et al [9] where high demand, low control and low support – concepts similar to alienation and tension in our study – predicted higher GHQ scores. Rather diminished personal accomplishment, which was independent of job stressors, predicted psychological morbidity. This finding suggests that individual differences – personality, motivation, attitudes, need for achievement, mental health history- rather than job-related variables alone are more likely to predict psychological morbidity. Furthermore, there is an indication that substance misuse practice involves psychological demands that are different from workload, dealing with complex patients, etc. These demands may include the practitioner's feeling of self-worth, role adequacy and personal achievement, which are often associated with opportunities to develop new skills and the use of a variety of skills [22,23].

### Limitations

There are limitations of this study that are mainly linked to the study design and sample. Firstly, the study adopted a cross-sectional design, which prevented conclusion regarding causality. A longitudinal design is better able to determine the causal relationship between job-related factors, burnout and psychological morbidity. McManus et al [10] have demonstrated the usefulness of longitudinal designs in burnout studies. Secondly, it was difficult to exclude the influence of social desirability that is often associated with self-administered questionnaire surveys. However, this is a limitation shared with many other studies of burnout and psychological morbidity. Thirdly, the reasons for non-response and the influence of age, gender and professional group on response rate were not examined. Consequently, it is plausible that the effect of non-response bias could have affected the results as non-responders may have differed in their experience of job stress, psychological morbidity and burnout. Another limitation is the age of the data, which may not reflect current patterns of psychological morbidity and burnout in the group studied.

### Study implications

Despite these limitations, the findings have provided useful information on job-related risks of burnout and psychological morbidity that can assist in the development of employee well-being programmes, and eventually enhance performance among substance misuse professionals. Furthermore, the findings can serve as a baseline for monitoring changes over time in the prevalence and pattern of burnout and psychological morbidity in the target group, by conducting repeated cross-sectional surveys in similar cohorts.

In terms of substance misuse practice, the findings should assist relevant policy makers in maintaining a healthy workforce. Firstly, there is evidence in this study that substance misuse professionals aged 25 years and below are at risk of emotional exhaustion. Therefore, there is a need for managers to provide adequate support for young practitioners who are likely to be new to the demands and challenges of substance misuse practice. Secondly, the strong association between personal accomplishment and psychological morbidity proves the need for employers to enhance staff competencies through professional development; this inevitably leads to improved self-esteem. Finally, the significant association between alienation, tension and the two burnout dimensions (EE and DP) suggests the need for employers to develop a work-based stress reduction programme that can assist substance misuse professionals in developing personal stress coping strategies.

Still, there are many unresolved questions about the relationship between demographic characteristics, job characteristics, job stressors and psychological morbidity. These include finding out the role of potential moderating variables such as personality, motivation, job attitudes, and mental health history. It is also possible that these variables are associated with burnout. These and other questions should be explored in future research on job stress among substance misuse professionals.

## Conclusion

In the sample of substance misuse professionals studied, rates of psychological morbidity and burnout were high, suggesting a higher level of vulnerability than in other health professionals. Furthermore, pathways to psychological morbidity and burnout are partially related. Therefore, targeted response is required to manage stress, burnout and psychological morbidity among substance misuse professionals. Such a response should be integral to workforce development.

## Competing interests

The author(s) declare that they have no competing interests.

## Authors' contributions

AO wrote the manuscript, analysed the data, interpreted the results and co-ordinated the study.

CC collected data, interpreted the results and revised the article for intellectual content.

RF was involved in study design, data interpretation and revising the article for intellectual content.

AO is the study guarantor.

This article is a product of the "Professionals Help Yourself" (PHY) programme being developed at St George's, University of London

## Pre-publication history

The pre-publication history for this paper can be accessed here:



## Supplementary Material

Additional file 1 Principal component analysis of job stressors with varimax rotation. The table describe three categories of job stressors – alienation, case complexity and tension. Click here for file

## References

[B1] National Treatment Agency for substance Misuse (2006). Models of care for treatment of adult drug misusers: Update 2006 London.

[B2] Elit L, Trim K, Mand-Bains IH, Sussman J, Grunfeld E (2004). Job satisfaction, stress and burnout among Canadian gynaecologic oncologists. Gynecologic Oncology.

[B3] Vanagas G, Bihari-Axelson S (2005). The factors associated to psychosocial stress among general practitioners in Lithuania. Cross-sectional study. BMC Health Services Research.

[B4] Cunrandi CB, Greiner BA, Ragland DR, Fisher JM (2003). Burnout and alcohol problems among urban transit operators in San Francisco. Addictive Behaviors.

[B5] Farmer R (1995). Stress and working with drug misusers. Addiction Research.

[B6] Knudsen HK, Ducharme LJ, Roman PM (2006). Counselor emotional exhaustion and turnover intention in therapeutic communities. Journal of Substance Abuse Treatment.

[B7] Lacoursiere RB (2001). "Burnout" and substance user treatment: The phenomenon and the administrator-clinician's experience. Substance Use & Misuse.

[B8] Avery AJ, Betts DS, Whittington A, Heron TB, Wilson SH, Reeves JP (1998). The mental and physical health of miners following the 1992 national pit closure programme. A cross-sectional survey using General Health Questionnaire GHQ-12 and Short Form SF-36. Public Health.

[B9] Calnan M, Wainwright D, Forsythe M, Wall B, Almond S (2001). Mental health and stress in the workplace: the case of general practice in the UK. Social Science and Medicine.

[B10] McManus IC, Winder BC, Gordon D (2002). The causal links between stress and burnout in a longitudinal study of UK doctors. Lancet.

[B11] SÖderfeldt M, SÖderfeldt B, Ohlson C-G, Thoerell T, Jones I (2000). The impact of sense of coherence and high-demand/low-control job environment on self-reported health, burnout and psychophysiological stress indicators. Work & Stress.

[B12] Karasek R, Brisson C, Kawakami N, Houtman I, Bongers P, Amick B (1998). The Job Content Questionnaire (JCQ). An instrument for internationally comparative assessments of psychosocial job characteristics. Journal of Occupational Health Psychology.

[B13] Calnan M, Wainwright D, Almond S (2000). Job strain, effort-reward imbalance and mental distress: a study of occupations in general medical practice. Work & Stress.

[B14] Maslach C, Jackson SE (1986). Maslach Burnout Inventory Manual.

[B15] Goldberg DP, Hillier VF (1979). A scaled version of the General Health Questionnaire. Psychological Medicine.

[B16] Farmer R, Clancy C, Oyefeso A, Rassool GH (2002). Stress and work with substance misusers: The development and cross-validation of a new instrument to measure staff stress. Drugs: education, prevention and policy.

[B17] Maslach C, Jackson SE, Leiter MP (1996). Maslach Burnout Inventory Manual.

[B18] Erens B, Primatesta P (1998). Health Survey for England.

[B19] De Vaus DA (2002). Surveys in social research.

[B20] Hosmer DW, Lemeshow S (2000). Applied logistic regression.

[B21] Karasek R (1979). Job demands, job decision latitude, and mental strain: Implications for job redesign. Administrative Science Quarterly.

[B22] Rafferty Y, Friend R, Landsbergis PA (2001). The association between job skill discretion, decision authority and burnout. Work & Stress.

[B23] Janssen PM, Schaufeli WB, Houkes J (1999). Work-related and individual determinants of the three burnout dimensions. Work & Stress.

