# Supplementary material for: Prevalence and associated factors in burnout and psychological morbidity among substance misuse professionals
Source: BMC Health Serv Res. 2008 Feb 8;8:39. doi: 10.1186/1472-6963-8-39 (PMC2265695; doi:10.1186/1472-6963-8-39)
Supplement: Additional file 1 — Principal component analysis of job stressors with varimax rotation. The table describe three categories of job stressors – alienation, case complexity and tension. [file 1472-6963-8-39-S1.doc]

**Additional file 1: Principal component analysis of job stressors with varimax rotation**

|  | **Factor** | | |
| --- | --- | --- | --- |
|  | 1 | 2 | 3 |
|  | **Alienation** | **Case complexity** | **Tension** |
| Lack of support from senior staff | **.724** | -.020 | .239 |
| Feelings of isolation | **.702** | .040 | .227 |
| Role ambiguity | **.630** | .152 | .347 |
| Decisions or changes which affect me are made 'above' | **.606** | .151 | .357 |
| Relationships with colleagues | **.578** | .267 | .178 |
| Poor quality of support staff | **.560** | .235 | .129 |
| Blurring of professional roles | **.528** | .297 | .346 |
| Unsupportive co-workers | **.527** | .159 | .176 |
| Lack of confidence in my management abilities | **.498** | .314 | .114 |
| Poor physical working conditions | **.476** | .096 | .262 |
| Uncertainty about the degree or area of responsibility | **.454** | .290 | .309 |
| Loss of one's professional identity | **.453** | .187 | .325 |
| Lack of management experience | **.431** | .272 | .178 |
| Difficulty in dealing with aggressive co-workers | **.423** | .312 | .168 |
| Relationships with subordinates | **.421** | .161 | .284 |
| Manipulative clients | .057 | **.819** | .209 |
| Demanding clients | .036 | **.775** | .264 |
| Dishonest clients | .038 | **.768** | .232 |
| Difficulty in dealing with passive clients | -.042 | **.733** | .324 |
| Hostile clients | .288 | **.709** | -.040 |
| Difficulty in dealing with aggressive clients | .351 | **.692** | -.010 |
| Frustration with clients' relapses | .023 | **.612** | .278 |
| Frustration with clients' lack of progress | .245 | **.606** | .259 |
| Dealing with adolescent clients | .048 | **.589** | .185 |
| Involvement with life and death situations | .279 | **.570** | .047 |
| Dealing with clients' relatives | -.014 | **.565** | .326 |
| Dealing with clients with overdose | .259 | **.555** | .033 |
| Coping with new situations | .341 | **.487** | .087 |
| Conflicting demands of my time at work by others | .310 | .008 | **.691** |
| Having too little time to do what is expected of me | .267 | .141 | **.676** |
| Conflicting demands between different job roles | .347 | -.050 | **.655** |
| Trivial tasks interfere with my job role | .245 | .155 | **.631** |
| Work overload | .226 | .182 | **.626** |
| Conflicts between my clinical and administrative roles | .260 | .213 | **.626** |
| Problems with allocating resources | .267 | .328 | **.622** |
| Conflict between my agency and others it must work with | .137 | .305 | **.533** |
| Shortage of essential resources | .369 | .027 | **.518** |
| Deciding task priorities | .253 | .266 | **.500** |
| Having to attend too many meetings | .173 | .221 | **.497** |
| Spending my time 'fighting fires' rather than working to a plan | .339 | .267 | **.495** |
| Organisational change | .260 | .151 | **.477** |
| Poor communication between my agency and other agencies | .199 | .284 | **.472** |
| Fluctuations in workload | .194 | .302 | **.462** |
